# Roles of TLR9: Gatekeeper to fungal infections

**DOI:** 10.1371/journal.ppat.1013185

**Published:** 2025-06-03

**Authors:** Elias Barbosa da Silva-Junior, Leonardo Freire-de-Lima, Luciana Polaco Covre, Alexandre Morrot, Herbert Leonel de Matos Guedes, Debora Decote-Ricardo, Celio Geraldo Freire-de-Lima

**Affiliations:** 1 Instituto de Biofísica Carlos Chagas Filho, Universidade Federal do Rio de Janeiro, Rio de Janeiro, Brazil; 2 Núcleo de Doenças Infecciosas, Universidade Federal do Espírito Santo, Vitória, Brazil; 3 Faculdade de Medicina, Universidade Federal do Rio de Janeiro, Rio de Janeiro, Brazil; 4 Instituto Oswaldo Cruz, FIOCRUZ, Rio de Janeiro, Brazil; 5 Instituto de Microbiologia Paulo de Góes, Universidade Federal do Rio de Janeiro, Rio de Janeiro, Brazil; 6 Instituto de Veterinária, Universidade Federal Rural do Rio de Janeiro, Seropédica, Brazil; Duke University School of Medicine, UNITED STATES OF AMERICA

## Introduction

Since Charles A. Janeway proposed in 1989 that the innate immune system possesses receptors with a remarkable ability to recognize “pathogen-associated molecular patterns”, which he termed “pattern recognition receptors” (PRRs) [1], significant advances have been made in understanding host–microorganism interactions. Toll-like receptors (TLRs) are classically described as an evolutionarily conserved family of proteins characterized by multiple leucine-rich repeats in their extracellular domain, while their cytoplasmic domain contains a unique Toll-interleukin (IL)-1 receptor (TIR) domain, homologous to the intracellular signaling domains of IL-1R and IL-18R. Janeway’s hypothesis was validated in 1996 when a study demonstrated that *Drosophila* was more susceptible to fungal infections when carrying mutations in receptors known as “Toll” [2]. The role of TLRs in microbial infections has long been investigated—including that of TLR9—resulting in significative advances in infectious diseases in the last two decades. In 2000, TLR9 was identified as a receptor capable of recognizing and responding to unmethylated CpG dinucleotides [3], and since then, its role in infections caused by viruses, bacteria, protozoa, and fungi has been extensively studied. However, the precise consequences of TLR9 deficiency or hyperactivation in response to various pathogens remain poorly understood. Although TLR9 is typically regarded as a receptor mediating inflammatory responses, its role in inflammation control during infection by certain pathogens warrants further discussion. Evidence suggests that its function may vary depending on the infecting pathogen and the immune cell type involved in the response [4–6]. Additionally, the role of TLR9 may differ based on its localization, which was once thought to be restricted to endosomal compartments. Several studies have now demonstrated the presence and function of TLR9 on the surface of various cell types, challenging the notion that ligand internalization into the cytoplasmic compartment is required for TLR9-mediated response [7]. Understanding how these mechanisms operate across different infections is critical to refining our perspective on TLR9-mediated immunity.

## A role beyond inflammation

TLR9 plays a crucial role in the development and function of plasmacytoid dendritic cells (pDCs) and B cell. pDCs act as sensors of the innate immune system, being particularly efficient in detecting viral nucleic acids and producing type I interferons [8]. During pDC development, TLR9 activation by pathogenic DNA or endogenous mitochondrial DNA (mtDNA) contributes to their maturation, differentiation, and functionality [7]. Additionally, TLR9 signaling influences the ability of pDCs to regulate inflammatory responses and interact with other immune cells, such as regulating the profile of T helper lymphocytes [8]. In the context of development of B cells, TLR9 influences the selection and survival of specific B cell subpopulations, including marginal zone B cells and regulatory B cells [9].

TLR9 is classically described as an endosomal receptor (eTLR9) capable of recognizing nucleic acids, although surface-expressed TLR9 (sTLR9) has been detected on certain immune, such as neutrophils and B cells, and non-immune cells, such as glial cells, where it plays an immunomodulatory role that remains largely enigmatic [7]. Like other members of the TLR family, TLR9 activation requires adaptor molecules harboring the TIR domain, such as myeloid differentiation primary response 88 (MyD88) and TIR-domain-containing adaptor protein inducing interferon-β (TRIF), ultimately leading to the transcription and secretion of cytokines, chemokines, and antimicrobial peptides (AMPs). It is natural to consider TLR9—along with the entire TLR family—as a receptor that triggers inflammation upon activation. However, studies on the regulatory role of TLRs in immune responses have been increasingly prevalent. The DNA of *Toxoplasma gondii* can activate TLR9, yet dendritic cells (DCs) do not become activated during infection in the absence of IFN-γ, and non-infected DCs serve as the primary producers of IL-12 in this context [10]. Apparently, TLR9 is partially responsible for an efficient Th1 antiparasitic response by regulating IL-12 production mediated by DCs and functions as a DNA sensor of the intestinal microbiota following tissue damage [10–13].

## TLR9-mediated inflammation: A double-edged sword?

The role of TLR9 in different cell types has been extensively studied in response to a wide range of infectious agents. Classically, TLR9 is expressed in human B cells, pDCs, monocytes, and macrophages, while in mice, it is expressed in macrophages and bone marrow-derived DCs (BMDCs) [7,8,14]. Upon ligand binding, TLR9 undergoes proteolytic cleavage and dimerization, enabling the recruitment of the adaptor protein MyD88. This interaction triggers a signaling cascade involving IRAK1, IRAK4, and TRAF3, leading to activation of downstream kinases such as IKKα. In pDCs, this pathway selectively engages the transcription factor IRF7, which translocates to the nucleus and drives the transcription of type I interferons (e.g., IFN-α and IFN-β) [15]. The production of type I IFNs is critical for antiviral immunity, promoting the activation of natural killer cells, enhancing antigen presentation, and establishing an antiviral state in neighboring cells [8,14,15]. However, the antifungal activity of type I IFN has also been demonstrated, as well as the importance of TLR9 in orchestrating a type I inflammatory response against fungal pathogens. TLR9 plays a crucial role in controlling *Cryptococcus* infection *in vivo* [16–19]. In addition, IFN-α/β are required to drive a protective Th1-type immune response during experimental infection with *Cryptococcus neoformans* [20]. Although type I IFN production via TLR9 activation is well described, IFN-α/β production through alternative pathways and the role of these cytokines in fungal infections remain to be fully elucidated. *Candida albicans* vesicles trigger type I IFN signaling independently of TLR9 [21]. Nevertheless, the regulation of type I IFN production and/or signaling appears to be important during fungal infection. It has been shown that *C. albicans* translocates TBK1 and abrogates type I IFN production via IRF3, resulting in downregulation of the type I inflammatory response [22].

The role of TLR9-mediated inflammatory response against pathogens is extensively studied but remains controversial. DCs are activated *in vivo* during the *Leishmania major* infection, and TLR9 appears to play a crucial role in this activation. *L. major* fails to activate DCs from TLR9-deficient mice. Furthermore, *L. major* DNA can activate DCs in a TLR9-dependent manner [23]. Evidence suggests that TLR9 is also significant for *Leishmania amazonensis* control. TLR9-deficient mice exhibit increased lesion size and parasitic burden, which is directly associated with a lower frequency of IFN-γ-producing CD8^+^ T cells during infection [5]. The fusion of TLR9 with the phagosome containing an obligatory intracellular parasite (*Leishmania*) could be critical for disease control. However, the role of TLR9 in *L. amazonensis* infection remains controversial, probably dependent of parasite strains. It has been demonstrated that DNA contained in *L. amazonensis* vesicles is responsible for activating TLR9, leading to increased expression of CD200 (which binds to the CD200R receptor), thereby inhibiting the inducible nitric oxide synthase signaling pathway and promoting the parasite’s intracellular survival [24].

**Fig 1:**
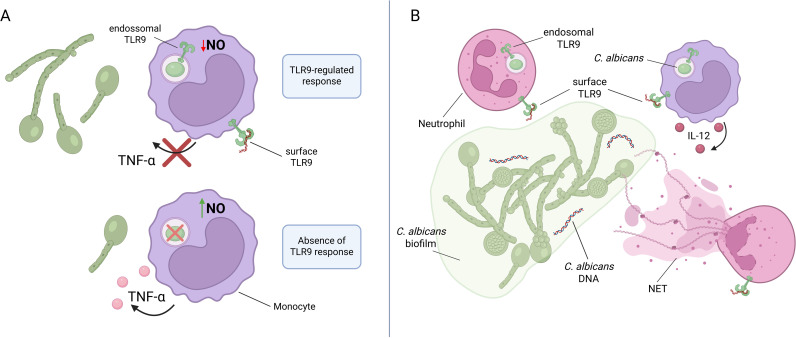
The dual role of TLR9 in *Candida albicans* infection. The role of TLR9 in the inflammatory response to *C. albicans* infection remains controversial. **(A)** Some studies suggest that TLR9 activation modulates the antifungal immune response by inhibiting TNF-α production, which may favor *C. albicans* intracellular survival. **(B)** Conversely, TLR9 activation induces IL-12 production by monocytes, promoting a protective Th1 antifungal response. Additionally, both endosomal TLR9 (eTLR9) and surface-expressed TLR9 (sTLR9) can recognize *C. albicans* DNA, contributing to neutrophil extracellular trap (NET) release for biofilm control. Both panels were created in BioRender. da Silva-Junior, E. (2025) https://BioRender.com/0nlnhoe.

Some studies have demonstrated that TLR9 is unable to initiate a proinflammatory response against *C. albicans* [6,25]. Interestingly, TLR9-deficient macrophages produce high levels of TNF-α and nitric oxide (NO), enhancing yeast killing—an effect absent when TLR9 is activated in WT macrophages [4] (Fig 1A). Although some studies have suggested that TLR9 does not play a significant role in the anti-*C. albicans* immune response and may even negatively modulate the inflammatory response [4,6], other studies have reported the opposite, leading to considerable controversy. *C. albicans* DNA has been detected during biofilm formation and was shown to trigger the release of neutrophil extracellular traps (NETs) [26]. TLR9 contributes to NET formation, but the underlying mechanisms are not yet fully understood. Although it is typically localized in endosomes, fully functional TLR9 capable of detecting DNA has been demonstrated on the surface of neutrophils [27]. *C. albicans* DNA can stimulate IL-12p40 production by BMDCs in a TLR9-dependent manner [28] (Fig 1B). Additionally, TLR9 plays a significant role for *C. neoformans* recognition, and the *URA5* gene has been shown to activate and stimulate IL-12p40 production by BMDCs in a TLR9-dependent manner [29].

TLR9 may cooperate with other innate immune receptors. It has been demonstrated that Dectin-1—which recognizes β-1,3-glucan, the major carbohydrate component of fungal cell wall—regulates the TLR9-dependent gene expression and controls the redistribution and accumulation of cleaved TLR9 in phagosomes containing *C. albicans* and *Aspergillus fumigatus* [30]. In addition, the inhibition of phagosomal acidification abrogates the TLR9 accumulation in the phagosomes containing β-1,3-glucan beads. However, it remains unclear whether this mechanism serves as an immune evasion strategy, with TLR9 acting as an immune response inhibitor, or whether TLR9 targeting to the phagosome is essential for fungal infection control.

## TLR9 as an important component of the barrier

**Fig 2:**
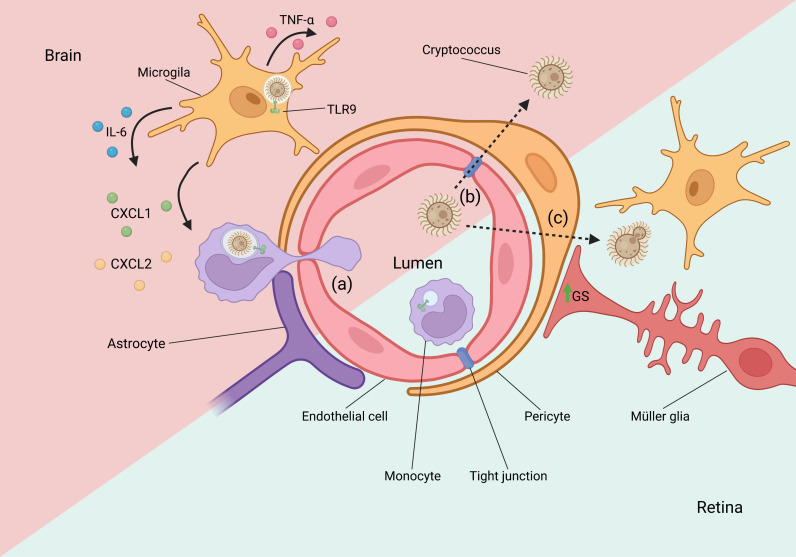
The role of TLR9 during epithelial-CNS barrier disruption by *Cryptococcus* spp. Cryptococcal yeasts can cross the blood–brain barrier (BBB) and blood-retinal barrier (BRB) by “Trojan horse” mechanism (a), paracellular pathway (b), and cellular traversal (c). *Cryptococcus* crosses the epithelial-CNS barrier after being carried by phagocytes that transmigrate to the brain, releasing the yeasts after exocytosis (lytic and non-lytic). Microglia can phagocyte cryptococcal yeasts and the TLR9-mediated antifungal immune response, characterized by TNF-α and IL-6 secretion. Chemotactic molecules CXCL1 and CXCL2 are secreted after TLR9 activation and promote monocytes and neutrophil recruitment, leading to a local inflammatory response. TLR9: Toll-like receptor 9; GS: Glutamine Synthetase; TNF-α: Tumor Necrosis Factor-α; CXCL: Chemokine (C-X-C) motif ligand; IL: Interleukin. Created in BioRender. da Silva-Junior, E. (2025) https://BioRender.com/4tapt8q.

Local immune responses against infections or other insults in the central nervous system (CNS) are often associated with glial cells. The notion that the CNS is immune-privileged, and that microglia and astrocytes exhibit macrophage-like characteristics leads us to consider these cells as crucial for controlling infections. The blood–brain barrier (BBB) and blood–retinal barrier (BRB) are composed of the neurovascular unit, which consists of an endothelial barrier and a glial barrier that together restrict the entry of pathogens and circulating immune cells [31]. Microglia and astrocytes are capable of regulating TLRs expression in response to different pathogens [32]. Among this, TLR9 is expressed in microglia and astrocytes, as well as in Müller glia, a retinal glial cell [33,34]. However, the role of TLRs in glial cells remains poorly understood, including the role of TLR9. Could be TLR9 important for maintaining selective permeability of epithelial-CNS barriers and/or controlling infections? The mechanisms by which certain microorganisms invade the brain have long been a subject of uncertainty. Recent studies suggest that *C. neoformans* rapidly penetrates the respiratory mucosa and crosses the BBB, followed by engulfment by microglia [35]. Moreover, the pulmonary environment—commonly the primary site of infection—is critical for triggering morphological changes in *C. neoformans* yeasts, facilitating their dissemination to extrapulmonary tissues such as the spleen and brain [36]. TLR9 activation induces the phagocytosis of *C. neoformans* yeasts and stimulates the secretion of TNF-α, IL-6, CXCL1, and MIP-2 (named as CXCL2) by microglia [37]. TNF-α and IL-6 are crucial for local inflammation against *C. neoformans* and for controlling of fungal dissemination to the brain [38,39], as well as the CXCL1 and CXCL2 important for monocytes and neutrophils recruitment to the site of infection. However, *C. neoformans* may exploit the phagocytic capacity of glial cells to disseminate or even cross epithelial barriers through evolutionarily conserved mechanisms [40,41]. Additionally, several studies have demonstrated that the “Trojan horse” mechanism is a key strategy for *C. neoformans* dissemination to the brain, highlighting the controversial role of phagocytosis in cryptococcosis [42,43] (Fig 2).

Similarly, TLR9 plays a crucial role during *Cryptococcus gattii* infection. TLR9-deficient mice exhibit a higher fungal burden in the brain compared to wild-type infected mice [16]. Furthermore, we recently demonstrated that the absence of TLR9 not only facilitates the entry of *C. gattii* yeasts into the brain but also their passage through the BRB. TLR9-deficient mice exhibit a high fungal burden, high deposition of GXM polysaccharide in the retina and retinal electrophysiology impairment, leading to visual loss during *C. gattii* infection [44]. In addition, Müller glial cells have increased expression of Glutamine Synthetase during retinal *C. gattii* infection, indicating intense activity of these cells in local inflammation, something absent in TLR9-deficient mice [44]. Apparently, TLR9 can recognize *Cryptococcus* prior to epithelial-CNS barriers disruption and may contribute to limiting yeast invasion. However, *Cryptococcus* utilizes multiple mechanisms to evade immune cellular detection and traverse epithelial barriers independently of the TLR9 response.

The cooperative action of TLR2 and TLR9 appears to be essential for controlling certain infections. In a *Trypanosoma brucei* infection model, TLR2/TLR9-deficient mice exhibited a higher parasite burden in the brain—a phenomenon observed in the absence of TLR9 but not observed in the absence of TLR2 only. Furthermore, the simultaneous deficiency of TLR2 and TLR9 drastically reduced the frequency of CD4^+^ and CD8^+^ T lymphocytes, as well as the expression of IFN-γ, IFN-β, and TNF-α mRNAs. These findings suggest that TLR9 also plays a crucial role in maintaining BBB integrity through cooperative immune mechanisms [45]. The cooperation between TLR2 and TLR9 leads to IL-6 and IFN-α/β production, protecting C57BL/6 mice by reducing brain viral load in a *Herpes Simplex Virus* infection model [46]. Recognition by TLR2/TLR9 is essential for controlling viral load in the brain and for the activation and recruitment of monocytes and natural killer cells [47]. However, the role of TLR9 in a Japanese Encephalitis Virus (JEV) infection model is not protective. mtDNA released following JEV-induced damage triggers TLR9 activation. This is associated with an increased population of myeloid-derived suppressor cells and a consequent inability to mount effective antiviral responses, leading to greater brain damage and higher mortality rates [48].

## Concluding remarks

TLR9 plays a pivotal and multifaceted role in host immunity, extending beyond its classical inflammatory responses that induces the control of microorganism. Its involvement in the development and function of immune cells, including pDCs and B cells, highlights its importance to immune homeostasis by inflammatory response regulation. In addition, the contribution to the integrity of BBB and BRB puts TLR9 as a crucial PPR. Its function in maintaining epithelial-CNS barrier integrity suggests a broader role in host defense. The interplay between TLR9 and other innate immune receptors, such as TLR2 and Dectin-1, demonstrates cooperative mechanisms that may enhance pathogen clearance. Moreover, its capacity to regulate immune responses in pathogen-specific and cell-type-dependent manners underscores its complexity as both a pro-inflammatory and immunomodulatory receptor. Despite extensive research, the precise consequences of TLR9 activation remain incompletely understood. Its role in infections, such as those caused by *C. albicans* and *L. amazonensis*, remains controversial, with evidence suggesting both protective and detrimental effects.

Future research should focus on elucidating the diverse functions of the TLR9 activation and cooperation, particularly in the context of infections that exploit its signaling pathways. A deeper understanding of its regulatory mechanisms, cooperative interactions with other PRRs and the differences (or similarities) between eTLR9 and sTLR9 could provide valuable insights for therapeutic strategies aimed at modulating immune responses in infectious diseases.
